# Whole-Organ Pancreas and Islets Transplantations in UK: An Overview and Future Directions

**DOI:** 10.3390/jcm12093245

**Published:** 2023-05-01

**Authors:** David Martin, Piero Alberti, Nicolas Demartines, Melanie Phillips, John Casey, Andrew Sutherland

**Affiliations:** 1 Department of Visceral Surgery and Transplantation, University Hospital CHUV, University of Lausanne (UNIL), 1015 Lausanne, Switzerland; demartines@chuv.ch; 2 Edinburgh Transplant Centre, Royal Infirmary of Edinburgh, Edinburgh EH16 4SA, UKmelanie.b.phillips@nhslothian.scot.nhs.uk (M.P.); john.casey@nhslothian.scot.nhs.uk (J.C.); andrew.sutherland@nhslothian.scot.nhs.uk (A.S.)

**Keywords:** pancreas transplantation, islets transplantation, type 1 diabetes mellitus

## Abstract

Whole-organ pancreas and islets transplantations are two therapeutic options to treat type 1 diabetic patients resistant to optimised medical treatment in whom severe complications develop. Selection of the best option for β-cell replacement depends on several factors such as kidney function, patient comorbidities, and treatment goals. For a patient with end-stage kidney disease, the treatment of choice is often a simultaneous transplant of the pancreas and kidney (SPK). However, it remains a major surgical procedure in patients with multiple comorbidities and therefore it is important to select those who will benefit from it. Additionally, in view of the organ shortage, new strategies to improve outcomes and reduce immune reactions have been developed, including dynamic organ perfusion technologies, pancreas bioengineering, and stem cell therapies. The purpose of this article is to review the indications, surgical techniques, outcomes, and future directions of whole-organ pancreas and islets transplantations.

## 1. Introduction

Diabetes mellitus (DM) is characterised by persistent hyperglycaemia which results from defects in the secretion or action of insulin. Type 1 diabetes results from the autoimmune destruction of the β-cells of the pancreatic islets and type 2 diabetes is caused from impaired insulin secretion and resistance to the action of insulin [1]. In 1921, Frederick Banting and Charles Best isolated insulin from pancreatic islets and administrated it to patients suffering from type 1 diabetes, thus inaugurating a new era in diabetes treatment [2]. Further developments, including synthetic short- and long-acting insulin, portable blood sugar monitoring and insulin pumps, have all improved diabetes management [3]. Despite this progress, a significant proportion of patients will suffer from the secondary complications of diabetes, such as retinopathy, neuropathy, nephropathy, and cardiovascular disease. Up to 60% of patients with end-stage kidney disease (ESKD) have DM, and those on dialysis have a 20% mortality rate within one year of initiation and a five-year survival rate under 50% [4]. Kidney transplantation may increase survival rates when compared to diabetic patients on dialysis [5]. However, poor glycaemic control after kidney transplantation remains an important issue and contributes to persistent morbidity and mortality [6,7]. 

Transplantation of the pancreas, either as a solid organ or as isolated islets of Langerhans, provides effective glycaemic control in type 1 and selected type 2 diabetic patients by replacing β-cells function. The first successful pancreas transplant was carried out by Kelly in 1966 [8]. Afterwards, the results of pancreas transplantation have continuously improved due to advances in surgical techniques and immunosuppressive therapy [9]. The first allogeneic islet transplants were reported from 1980, but the first 20 years of application have shown a lack of regularity and accuracy in the benefits, with overall success rates of around 10% [10,11,12]. Simultaneous pancreas and kidney (SPK) transplant is now the treatment of choice for patients with type 1 diabetes and diabetic nephropathy. However, SPK is a major procedure in patients who often have multiple comorbidities. There are other treatment options, including simultaneous islet kidney transplant (SIK), pancreas after kidney transplant (PAK), and islet after kidney transplant (IAK) in patients with kidney disease, or pancreas transplant alone (PTA) and islet transplant alone (ITA) in patients without kidney disease.

This review focuses on the indications, types of transplantation, organ allocation, surgical techniques including procurement, and postoperative outcomes. Moreover, it summarizes recent progress and future directions needed in order to improve patient safety and outcomes.

## 2. Epidemiology

As of 2020, more than 63,000 whole-organ pancreas transplants had been performed worldwide, including 35,000 in the USA [13]. There are 105 centres in the USA that perform approximately 1000 pancreas transplants per year [13,14]. In the UK, around 180 pancreas transplants are performed each year in eight centres [15]. Between 80 and 90% of solid pancreas transplants are performed as part of an SPK, the remaining cases are isolated pancreas transplants (PTA or PAK) [15,16]. Concerning islets, a recent international survey focusing on program activities in the 2000–2020 period, 4365 islet allotransplants were reported in 2170 patients (2608 in Europe, 1475 in North America, 135 in Asia, 119 in Oceania, and 28 in South America) [17]. Around 30 islet transplants are performed per year in seven centres in the UK [15]. Donor pancreases are retrieved from around the country and sent to the one of the three isolation facility for processing, before the islets are distributed for transplantation at the recipient’s transplant centre [18]. The majority of cases carried out are ITA (81%), with IAK occurring less often (17%), and only a small number of SIK transplantation per year. 

## 3. Assessment, Transplant List, and Allocation

The involvement of a multidisciplinary team is required in the assessment of a patient, and may involve different specialists, including physicians, surgeons, anaesthetists, radiologists, transplant co-ordinators, dieticians, and, if indicated, psychiatrists. There are three stages of assessment: pre-transplant assessment, decision, and listing. Usually, the pre-transplant assessment should be completed within 18 weeks of referral, and usually includes diabetic condition, social and past medical history, radiological investigations (ultrasound of the liver, +/− doppler of aorta and iliac arteries), dental and cardiac assessment, haematology and biochemistry tests, serology for viruses and immunology tests (HLA) [19]. Additional studies may include oral or intravenous glucose challenge, anti-insulin, anti-glutamic acid decarboxylase and islet cell antibodies, proinsulin level and lipoprotein. Then, the decision will be made as to whether the patient should be listed for transplantation, according to the assessment, the multidisciplinary consensus, and the selection criteria (described in the following sections). Absolute contraindications to pancreas and islets transplantation include excessive cardiovascular risk (non-correctable coronary artery disease or myocardial infarction within six months), non-curable malignancy, active sepsis, major psychiatric history likely to result in non-adherence, and inability to withstand surgery and immunosuppression [19]. Each listing decision, whether for the pancreas or the islets, is therefore made after multidisciplinary consensus, and after a first meeting with the patient. Indeed, the patient’s perspective and preference should be considered in preoperative discussions. They comprise an interplay of physical, psychological, and social factors that should be addressed when planning a pancreas or islets transplant. 

In the UK, pancreas and islets are on a shared allocation list, while in many other countries, islets are isolated from pancreases that are declined for whole-organ transplantation [20]. Seven factors are included in a national allocation scheme, and include cold ischemic time, sensitization to HLA, dialysis status, waiting time, donor-recipient age matching, donor BMI, and donor-recipient HLA mismatch [21]. Each factor is given a different points-weighting, and the patient on the waiting list with the highest points will be offered the transplant first.

At the end of 2021, there were 299 patients on the UK active pancreas and islet transplant list [22]. The median waiting time for patients registered for a pancreas transplant is 352 days (95% confidence interval (CI) 326–378 days), and 631 days (95% CI 338–924 days) for an islet transplant (including simultaneous islet and kidney) [22]. One issue to consider is the lack of consistent referral of transplant candidates from endocrinologists [23].

## 4. Indications and Current Practice

**Figure 1:**
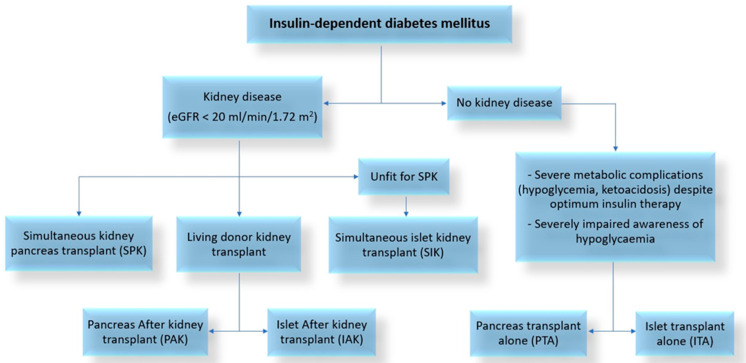
Types of pancreas and islet transplant.

Islets and pancreas transplants are mainly offered to patients with type 1 diabetes. Less frequently, transplants are performed for type 2 diabetes (pancreas only), and less common forms of DM, including cystic fibrosis-related diabetes (CFRD), and post-pancreatectomy or post-pancreatitis DM, in which transplantation can reverse both endocrine and exocrine insufficiency [19,24,25]. Whether a whole-organ pancreas or islets are transplanted depends, in part, on clinical accessibility to these procedures and patient’s comorbidities and goals, as both are effective treatments (Table 1) [26]. Islet transplant is associated with a low rate of complications related to the procedure and is highly successful at eliminating severe hypoglycaemia and glucose lability [26]. However, more than one transplant may be needed to achieve insulin independence [27]. Pancreas transplant exposes the patient to a higher risk of intra- and post-operative complications but has a high likelihood of resulting in insulin independence after a single transplant [26]. Pancreas and islet cell transplant options are provided in Figure 1, and a summary of the indications, contraindications, and choice of type of transplants is described in Table 2.

### 4.1. Simultaneous Pancreas and Kidney (SPK) Transplant/Simultaneous Islet Kidney (SIK) Transplant

SPK is the treatment of choice for patients with type 1 diabetes and diabetic nephropathy receiving dialysis or with an estimated glomerular filtration rate < 20 mL/min/1.73 m^2^. SPK recipients have better kidney allograft and patient survival compared to deceased-donor kidney transplant or living-donor kidney transplant alone [4]. Transplantation can also be considered in insulin-dependent type 2 diabetic patients with renal failure who have a body mass index < 30 kg/m^2^ with low insulin requirements (<1 U/kg of body weight) [3,28]. The decision in these patients is whether to undergo a kidney transplant alone with subsequent pancreas transplant instead of a combined operation. Long-term graft function remains highest in patients receiving a pancreas through SPK, compared to PAK or PTA patients [27]. However, several factors must be taken into consideration, such as availability of potential living donor, the urgency to come off dialysis, and waiting time for an SPK. One of the advantages of SPK is that it can directly improve diabetic outcomes in patients who already require major surgery and immunosuppression for renal transplant.

For patients who meet the listing criteria for SPK but who have been deemed unsuitable for this major surgery, SIK is a treatment option that has the immunological advantages to receive islets from the same deceased donor. This rarely performed procedure provides results comparable to pancreas transplantation in terms of glucose control and avoidance of severe hypoglycaemia, but insulin independence rates are significantly lower after one year (31% vs 96% in SPK group) [29].

### 4.2. Pancreas Transplant Alone (PTA)/Islet Transplant Alone (ITA)

PTA or ITA is recommended for type 1 diabetic patients with frequent severe metabolic complications despite optimal insulin therapy, and if their renal function is normal. According to the Pancreas Advisory Group on behalf of NHS Blood and Transplant, patients must have had at least two severe hypoglycaemic episodes within the last 24-months [19]. Severely impaired awareness of hypoglycaemia is another criterion, and the American Diabetes Association (ADA) defines an episode as “an event requiring the assistance of another person to actively administer carbohydrate, glucagon, or other resuscitative actions”. The number of PTA and ITA remain low compared to SPK and SIK, respectively, although the results seem encouraging when compared to insulin treatment. A recent trial comparing the metabolic efficiency of islet transplantation to insulin therapy for type 1 DM treatment (TRIMECO trial) confirmed a clear benefit of islet transplantation [30]. ITA seem superior to medical therapy in resolving severe hypoglycaemia and preventing the progression of microvascular complications [30,31]. The choice between PTA and ITA is outcome-driven (awareness vs. insulin independence) and depends on local practices and policies. While PTA requires a single donor, ITA requires several donors, which increases waiting-list time. Furthermore, islet processing is effective in producing transplantable islets in 50–89% of the pancreases processed, and one to four infusions are required to achieve the desired clinical effects [31,32]. In terms of clinical outcomes, insulin independence is achieved promptly after PTA, whereas, it is delayed, sometimes by months after ITA, depending on the number of transplants and the success of engraftments. Recovery from hypoglycaemia unawareness, with or without exogenous insulin, and graft survival are however similar between PTA and ITA [32]. Potential candidates for ITA are the elderly, frail, and morbid patients suffering from labile type 1 DM with severe hypoglycaemia [26]. Other key points to consider are the baseline clinical condition of the recipient as well as their preference and perception of risks and benefits for each procedure.

### 4.3. Pancreas after Kidney (PAK) Transplant/Islet after Kidney (IAK) Transplant

Patients who match the criteria for PTA or ITA and have already received a living or deceased kidney transplant may be considered for PAK or IAK, respectively. PAK transplantation has historically been associated with inferior pancreas allograft survival compared with SPK transplantation, because of higher rates of thrombosis and immunologic graft loss [33,34]. More recently, technical failure rates have dropped to 5%, and pancreas allograft survival in PAK has improved, with, respectively, 85% and 60% of patients being insulin-free at one-0year and five years [34]. A PAK exposes the patient to more surgical risk but generally provides higher endocrine reserve compared to the alternative option of IAK transplantation, which is usually proposed for patients considered not fit enough for major surgery [35]. An unresolved aspect of PAK and IAK is optimal timing for the transplant after a successful kidney transplant.

## 5. Pancreas Retrieval

The standard pancreas graft includes the entire pancreas, a duodenal segment, and the spleen en bloc. Arterial supply is provided by the splenic artery and the superior mesenteric artery (SMA), while venous drainage is provided by the splenic and portal vein [36]. An arterial “Y iliac graft” is procured from the bifurcation of the donor common iliac artery for future vascular reconstruction. Approximately 50% of all deceased donor pancreases procured for the purposes of either pancreas or islet transplantation are discarded each year in the UK [37]. The most frequent reasons for organ discard are insufficient islet yield (19%), fatty organ (17%), miscellaneous reasons (17%), and organ damage (10%). The pancreas is a challenging organ to procure, and higher rates of damage are reported than for either liver or kidney retrievals [15]. It is also important to note that the procurement must be carried out in the same way as for an organ or islet transplant. Careful training, supervision, and meticulous surgical approach are required to minimise organ damage.

Since the first Maastricht category III Donation after Circulatory Death (DCD) donor pancreas transplant was performed in 2005, there has been a rapid expansion of this program in the UK, which now accounts for approximately 25% of the pancreas transplants performed each year [15]. It has been shown that that 1 year pancreas and patient survivals were similar between DCD and Donation after Brainstem Death (DBD) [38]. Similar results were found in the USA, with comparable graft survival at 1, 3, 5, and 10 years [39]. Concerning islets transplantation, approximately 10% of them come from controlled DCD donors [15]. The published experiences from DCD donors for islet transplantation is limited, but early results suggest reasonable outcomes [40]. In DCDs, organs are exposed to a period of warm ischemia, which is thought to have a detrimental effect on organ quality [41]. However, there is no consensus when warm ischemia starts and how long a pancreas can sustain it before becoming irreversibly damaged. Current UK guidelines suggest abandoning pancreas retrieval if the functional warm ischaemic time (defined as donor systolic BP < 50 mmHg) is more than 30 min [42].

## 6. Transplant Procedures

**Figure 2:**
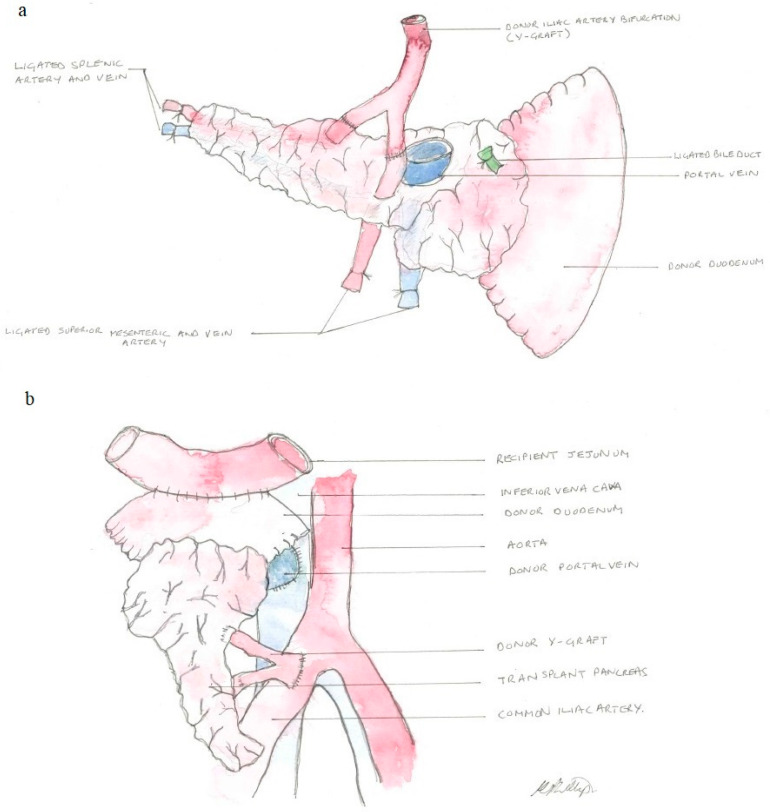
Surgical technique of whole-organ pancreas transplantation: (**a**) back-table preparation of pancreas graft. The segment of donor duodenum is shortened by additional stapling and reinforced with sutures, the spleen is removed, and peripancreatic tissue ligated and removed. The arterial supply via the superior mesenteric and splenic arteries is connected to a Y graft from the bifurcation of the donor common iliac artery in order to provide a single arterial inflow. Venous outflow through the portal vein; (**b**) the duodenum is anastomosed to the recipient’s jejunum for enteric drainage. Arterial supply is achieved by anastomosis to the common iliac artery, and venous drainage to the inferior vena cava.

Before whole-organ pancreas transplantation, back-table preparation is crucial in to improve postoperative outcomes (Figure 2). After removal of the spleen, the arterial supply via the superior mesenteric and splenic arteries is attached to the “Y graft in order to provide a single arterial inflow that is anastomosed to common or external iliac artery from the recipient [3,43]. The portal vein is anastomosed to the recipient inferior vena cava, iliac veins, or portal vein, and the donor duodenum is anastomosed to recipient duodenum or small bowel [36]. A United Network for Organ Sharing (UNOS) registry study showed that there was no significant clinical difference in patient or allograft survival between portal or systemic venous drainage in pancreas transplantation [44]. In PTA, the duodenum can also be anastomosed to the bladder, it allows monitoring of urinary amylase for diagnosis of rejection [45]. However, this exocrine drainage is rarely used, due to the high rate of urological and metabolic complications [46]. Pancreas grafts are placed either intraperitoneally or extraperitoneally in the right side of the pelvis due to the favourable disposition of the right iliac vessels [36]. In case of SPK, kidney grafts are placed on the contralateral iliac fossa, either intraperitoneally or extraperitoneally [47,48]. Insofar as there is only very little prospective comparative data, and that the techniques are multiple, the choice of a surgical method should remain the preference of the surgeon.

Concerning islets, they are isolated from donor pancreas by collagenase digestion [11]. Islets are purified to remove exocrine fragments. It is recommended to have at least 5000 islet equivalent units (IEQs)/kg body weight of recipients, but on average, about 350,000 to 400,000 IEQs are transplanted in each procedure [49,50]. These purified islets (containing beta, alpha and delta cells) are transplanted by percutaneous transhepatic access into the portal vein under radiological guidance. Subsequently, islets engraft into the liver of the recipient [26]. A summary of the technical challenges and the postoperative risks is provided in Table 1. 

## 7. Immunosuppression

Since approximately two decades, predominant immunosuppression protocol for pancreas and islets transplantation includes of induction therapy with a T cell-depleting agent (antithymocyte globulin or a monoclonal antibody such as alemtuzumab) and maintenance therapy using a calcineurin inhibitor (tacrolimus) and mycophenolate mofetil [26]. A recent review from 2020 review confirmed the wide use and the clinical efficacy of this regimen [51]. Many centres do not use corticosteroids or plan for early withdrawal. However, there is currently no gold standard immunosuppression regimen universally accepted and applied. Immunosuppression modalities are a very dynamic field of research, and new therapeutic strategies and combination protocols to reduce or avoid drug toxicities and immune-related complications should improve outcomes after pancreas or islets transplantation in the coming years [52].

## 8. Outcomes

Whole-organ pancreas transplantation is a major procedure that is associated with significant postoperative morbidity, such as graft arterial or venous thrombosis (10–35%), enteric leakages with subsequent infections and fistulae, pancreatitis, and bleeding (5–10%). Intestinal bleeding is a frequent complication of enteric drainage, and usually originates from suture line, ischemic duodenal ulcers, or duodenitis [53]. Other complications of enteric drainage are anastomotic leaks leading to sepsis, and intestinal obstruction [36]. Unfortunately, these complications result in a relaparotomy rate of 20–30% and a graft pancreatectomy in up to 10–15% of recipients [54]. Rejection rates are in the range of 5–25% depending on what immunosuppressive regime is used [55]. A clear distinction between surgical and immunologic complications is not always possible as they can be triggered by each other [36]. Vascular thrombosis and enteric leaks can be caused by acute rejection, whereas bleeding can complicate chronic graft rejection or viral infections [56,57]. Therefore, refinements in surgical outcomes have often been preceded by improvements in graft preservation, immunosuppression, and antimicrobial prophylaxis [45,58]. A study from the International Pancreas Transplant Registry (IPTR) revealed that the one-year patient survival reached over 95%, and over 83% after five years [59]. In this study, the one-year immunological graft loss rate was 1.8% in SPK, 3.7% in PAK, and 6.0% in PTA. Insulin independence is obtained immediately after whole pancreas transplantation and persists within years in almost 60–70% of cases, and this translates into clinical improvements with respect to diabetic nephropathy, neuropathy, gastroparesis, retinopathy, and cardiac function [60].

Islet transplant is associated with a lower rate of complications related to the procedure [26]. The most common complications include bleeding, thrombosis, and iatrogenic injury to anatomical structures, with an overall incident risk reported of less than 10% [61]. Glucose stability is obtained within days to weeks, but insulin independence (if it is achieved) is delayed for several months, as the isolated islets must engraft and revascularise in the new liver environment before reaching full functionality [62]. Islets transplantation also usually requires more than one transplant to achieve insulin independence [27]. A large multicentre trial showed that 87% of the patients achieved complete resolution of severe hypoglycaemia at one year, while only 52% were insulin independent [62]. It has been shown that islet transplantation with the use of the Edmonton protocol could successfully restore long-term endogenous insulin production and prevent severe hypoglycaemia in subjects with type 1 DM, but insulin independence was usually not sustainable [63,64].

## 9. Recent Progress and Future Directions

### 9.1. New Technologies and Organ Preservation

There is still a disparity between transplant demand and donor availability. In this context, the transplant community has tended to suggest the use of extended-criteria donors, such as older donors with comorbidities and DCDs. This approach may be accompanied by higher susceptibility to the ischemia-reperfusion injury, insofar as the warm ischemia time is prolonged [65]. Graft pancreatitis and thrombosis, arising from ischemia reperfusion injuries, are major causes of graft loss [66]. There are several solutions for perfusion and preservation of the pancreas during and after procurement, such as University of Wisconsin (UW), histidine–tryptophan–ketoglutarate (HTK), Celsior, or Institut Georges Lopez-1 (IGL-1). A systematic review including 939 transplants showed that the different cold perfusions had comparable results in terms of graft pancreatitis, thrombosis, and graft survival [67].

Static cold storage is considered the standard method of preservation, but dynamic techniques such as machine perfusion (both hypothermic and normothermic) have progressed rapidly over the past two decades. A systematic review of human and animal’s experimental studies showed that ex situ pancreas perfusion reduced ischemic reperfusion injuries, pancreas thrombosis, morbidity of transplantation, and could be a helpful tool to evaluate pancreas prior to transplantation [66]. Normothermic ex situ perfusion with supplemental oxygen replicates near physiological parameters, and seems to be an interesting alternative promising strategy for pancreas organ preservation [68]. However, ideal perfusion parameters are difficult to establish in pancreas transplantation given its complex vascular anatomy and blood flow.

In normothermic regional perfusion (NRP), warm oxygenated blood is recirculated in the donor (in situ) and is now an established practice in several European countries even if it is a technically and logistically challenging procedure. NRP was initially introduced to recondition and assess the liver in order to decrease ischemic cholangiopathy rates in liver transplantation. However, the use of NRP during DCD organ recovery led to increased organ utilization and improved transplant outcomes in kidney and pancreas transplantation as well [69]. The procedure involves placing the donor onto a cardio-pulmonary bypass circuit after the declaration of death, with an aortic balloon or clamp used to prevent reperfusion of the brain or heart [40]. Promising results from DCD organs utilised after NRP have been shown by the Edinburgh team [70]. In a national retrospective study of UK DCD donors, this procedure was associated with improved organ utilization rates for pancreas from the total number of organ offers, but 12-month transplant survival was comparable for recipients who received a DCD NRP graft compared with those who did not [69]. 

Novel perfusion and preservation technologies are an exciting innovation in pancreas transplantation, but the clear benefits in terms of graft function and clinical outcomes are still too little studied. Future investigations are required in order to standardize management and generated the initial outcome data [70]. Furthermore, implementation of these perfusion technologies is logistically complex, require training, and are expensive.

### 9.2. Advanced Therapies

The shortage of donor pancreas’ is one of the greatest challenges facing the field of organ transplantation. In this context, advanced therapies have been developed to replace lost β-cells, including stem cell therapies and islets engineering strategies [26,71]. These treatments may have the potential to reduce or eliminate the need for immunosuppression. For example, islets grafts can be encapsulated before transplantation, which creates a barrier against large immune cells and cytotoxic molecules, thus avoiding rejection while still allowing the active diffusion of oxygen, glucose, nutrients, and hormones through a membrane [71,72,73]. There are two possibilities; microencapsulation, in which islets are individually encapsulated in alginate gel spheres (1 mm), or macroencapsulation, in which islets are encapsulated in devices that are anastomosed to the vascular system or implanted into subcutaneous soft tissues [74]. Encapsulated islets have been successfully transplanted into the peritoneal cavity and under the skin, both in animal models and humans, however with moderate success [75,76,77]. Poor oxygen diffusion and pericapsular fibrosis seem to be recurrent issues that result in high failure rates [72,78]. Clinical trials in human subjects are currently in progress.

Endocrine pancreas bioengineering is thus an emerging field in β-cell replacement, which might provide endocrine cells with all the building blocks (vascularization, extracellular matrix, architecture) useful for their successful engraftment and function in vivo [79]. An international group has recently developed a Neovascularized Implantable Cell Homing and Encapsulation (NICHE) device, which integrates direct vascularization for facile mass transfer and localized immunosuppressant delivery for islet rejection prophylaxis [80]. This new treatment modality was tested in rats, and authors concluded that it was a promising approach for safe and effective islet transplantation and long-term type 1 DM management.

Human pluripotent stem cell-derived islets were produced in different laboratories worldwide and studied in animal models, with some promising results and a potential cell resource for diabetes treatment [81,82]. However, these new approaches have not been systematically assessed in humans or animal models physiologically similar to humans [80]. Xenogeneic islets isolated from pigs is another area of research. Trials of porcine islet xenografts transplanted into primate models have shown a potential to achieve insulin independence and prolonged islet-graft function (>600 days) [34,35]. The disadvantage is that it seems to cause a greater immunological hurdle compared with islet allotransplantation [26,83].

These efforts in engineering, regenerative medicine, and xenogeneic islets from pigs will meet some issues concerning their clinical application in humans and ethical concerns regarding their origin, as well as how, when, and where will patients have access to these advanced therapies. There are several dangers associated with xenotransplantation, such as immunological rejection of the organ, endogenous viruses infecting the recipients, animal welfare, and fair distribution of organs [84]. With a shortage of human tissue available, it is no wonder that researchers have considered the pig as a potential source. For example, porcine valves are routinely used in aortic valve replacements in the UK. However, there is currently no legislation which specifically regulates xenotransplantation in the UK, but a non-statutory body, the UK Xenotransplantation Interim Regulatory Authority (UKXIRA) exists to monitor developments in the xenotransplantation field.

The lack of uniform definitions for success in β-cell replacement therapy should be addressed in order to make treatment options comparable in the future [26]. A recent survey of type 1 DM and their family members showed that more than 90% of them wished to be insulin-free and have stable glycaemic control, and 90% would accept a new treatment modality, including islets engineering and stem cell therapy [85]. While quality of life may improve following pancreas or islets transplantations, patients may still experience psychological distress (e.g., anxiety, concerns about future health), physical changes (e.g., weight gain), and social challenges (e.g., inability to work) [86,87]. Patient-reported outcome measures (PROMs) are useful for patient management, as they are more sensitive to outcomes relevant to the patients themselves, compared to the traditional outcome parameters, such as length of hospital stay or complications. A qualitative study compared the PROMs of transplanted SPK patients versus those still waiting [87]. SPK recipients showed improved treatment satisfaction, well-being, self-reported health, and generic quality of life, while diabetes complications, self-imposed blood glucose monitoring and dietary restrictions continued to impact quality of life negatively post-transplant [87]. Another dimension to consider is the patient-reported experience measures (PREMs) that assess experiences with health care from the patient’s perspective. An interesting qualitative study examined PREMs in SPK transplant recipients and showed that the most important categories were receiving sufficient information prior to the intervention and the waiting time for transplantation and its consequence [88]. The patients also highlighted the importance of having rough estimates for the transplantation date to better organize their personal and work life, and the need to better manage the distress caused by the waiting time for transplantation or the last-minute cancellation of their surgery [88]. Administrative barriers, communication, and transfer of medical between hospitals, as well as patient accessibility and logistics are other areas of concern that usually trigger anxiety in patients.

PROMs and PREMs are still too little studied in pancreas and islet transplantation and should be assessed in a repeated and standardised manner in the future to improve the global care of patients by sharing clinical decision. 

## 10. Conclusions

Whole-organ pancreas and islets transplantations are two therapeutic options to treat type 1 diabetic patients resistant to optimised medical treatment. Selection of the best treatment modality depends on several factors, such as kidney function, patient comorbidities, and treatment goals. Both approaches should be considered as complementary treatment options for β-cell replacement, rather than competing. However, they are limited by organ shortage. In this context, new strategies to improve outcomes and reduce immune reactions have been developed, including dynamic organ perfusion technologies, pancreas bioengineering, and stem cell therapies.

## Figures and Tables

**Table 1:** Clinical aspects of pancreas and islets transplantations.

Approach	Accessibility	Advantages	Disadvantages	Objectives
**Whole-organ pancreas**	- Recognised procedure	- High rate of insulin independence with one transplant - Effective in glucose control	- Major procedure; high morbidity risk	- Primary: insulin independence - Primary/secondary: eliminate hypoglycaemia
Techniques	- Back-table, donor arterial Y graft connected to donor SMA and SA, Y graft connected to recipient CIA - PV connected to recipient IVC, and donor duodenum to recipient small bowel			
Risk	- Graft arterial or venous thrombosis, bleeding, infections, enteric leakages, pancreatic fistulae, pancreatitis, intestinal, relaparotomy, graft pancreatectomy			
**Islets**	- Research protocol (USA) - Recognised procedure (Canada, Europe)	- Minor procedure - Effective in glucose control	- Delayed onset of function - More than 1 transplant needed for insulin independence	- Primary: eliminate hypoglycaemia - Secondary: insulin independence
Techniques	- Isolation facility: collagenase digestion, islets purified (5000 islet equivalent units/kg body weight) - Transplanted by percutaneous transhepatic access into PV under radiological guidance			
Risk	- Bleeding, portal vein thrombosis, and iatrogenic injury to adjacent organs			

**Table 2:** Recommendation for patient selection.

	SPK	SIK	PTA	ITA	PAK	IAK
Indication *	Type 1 DM with nephropathy (GFR < 20 mL/min/1.73 m^2^) Type 2 DM with nephropathy and BMI < 30 kg/m^2^	Type 1 DM with nephropathy (GFR < 20 mL/min/1.73 m^2^) When patients are unfit for SPK.	Type 1 DM, normal renal function, severe hypoglycaemic episodes and/or impaired hypoglycaemia awareness. The choice is outcome-driven (see Table 1), depends on patient comorbidities and patient’s fitness.		Patients who match the criteria for PTA or ITA and have already received a kidney transplant. The choice is outcome-driven (see Table 1), depends on patient comorbidities and patient’s fitness.	
**Contraindications**	Excessive cardiovascular risk, malignancy, active sepsis, major psychiatric history, inability to withstand immunosuppression.					
**Allocation factors**	Cold ischemic time (travel time), sensitization to HLA, dialysis status, waiting time, donor-recipient age matching, donor BMI, and donor-recipient HLA mismatch.					

SPK, simultaneous pancreas and kidney; SIK, simultaneous islet kidney transplant; PAK, pancreas after kidney transplant; IAK, islet after kidney transplant; PTA, pancreas transplant alone, ITA; islet transplant alone; DM, diabetes mellitus; GFR, glomerular filtration rate; BMI, body mass index. * Patients must be resistant to optimised medical treatment (insulin) and present metabolic complications.

## Data Availability

Not applicable.
